# Supplementary material for: Hypertrophic chondrocytes serve as a reservoir for marrow-associated skeletal stem and progenitor cells, osteoblasts, and adipocytes during skeletal development
Source: eLife. 2022 Feb 18;11:e76932. doi: 10.7554/eLife.76932 (PMC8893718; doi:10.7554/eLife.76932)
Supplement: Source code 1. [file elife-76932-code1.zip › Coding for single cell data generation.pdf]

Seurat v3 for 2M dataset

```
library(Seurat)
library(dplyr)
library(umap)
library(monocle3)
long <- Read10X("../seq/")
long<- CreateSeuratObject(long, min.cells = 5)
long[["percent.mt"]] <- PercentageFeatureSet(long, pattern = "^mt-")
VlnPlot(long, features = c("nFeature_RNA", "nCount_RNA", "percent.mt"), ncol = 3)
plot1 <- FeatureScatter(long, feature1 = "nCount_RNA", feature2 = "percent.mt")
plot2 <- FeatureScatter(long, feature1 = "nCount_RNA", feature2 = "nFeature_RNA")
CombinePlots(plots = list(plot1, plot2))
long <- subset(long, subset = nFeature_RNA > 200 & nFeature_RNA < 4000 & percent.mt < 7)
long <- NormalizeData(long, normalization.method = "LogNormalize", scale.factor = 10000)
long <- subset(long, subset = tdtomato > 2)
long <- FindVariableFeatures(long, selection.method = "vst", nfeatures = 2000)
top10 <- head(VariableFeatures(long), 10)
plot1 <- VariableFeaturePlot(long)
plot2 <- LabelPoints(plot = plot1, points = top10, repel = TRUE)
CombinePlots(plots = list(plot1, plot2))
all.genes <- rownames(long)
long <- ScaleData(long, features = all.genes)
long <- ScaleData(long, vars.to.regress = "percent.mt")
long <- RunPCA(long, features = VariableFeatures(object = long))
ElbowPlot(long, 50)
long <- FindNeighbors(long, dims = 1:25)
long <- FindClusters(long, resolution = 0.4)
long <- RunUMAP(long, dims = 1:25)
DimPlot(long, reduction = "umap")
```

Seurat v4 for e16.5 dataset

```
library(Seurat)
library(monocle3)
library(dplyr)
library(stringr)
library(SeuratData)
library(SeuratWrappers)
library(ggplot2)
library(patchwork)
library(magrittr)
s.genes <- str_to_title(cc.genes$s.genes)
g2m.genes <- str_to_title(cc.genes$g2m.genes)
long1 <- Read10X("../filtered_feature_bc_matrix/")
```

```
long<- CreateSeuratObject(long1, min.cells = 5)
long[["percent.mt"]] <- PercentageFeatureSet(long, pattern = "^mt-")
VlnPlot(long, features = c("nFeature_RNA", "nCount_RNA", "percent.mt"), ncol = 3)
plot1 <- FeatureScatter(long, feature1 = "nCount_RNA", feature2 = "percent.mt")
plot2 <- FeatureScatter(long, feature1 = "nCount_RNA", feature2 = "nFeature_RNA")
plot1+plot2
long <- subset(long, subset = nFeature_RNA > 200 & percent.mt < 15)
long <- NormalizeData(long)
long <- subset(long, subset = ai9tdTomato > 2)
long <- CellCycleScoring(long, s.features = s.genes, g2m.features = g2m.genes, set.ident =
TRUE)
long <- SCTransform(
  long,
  assay = 'RNA',
  new.assay.name = 'SCT',
  vars.to.regress = c('percent.mt', 'nFeature_RNA', 'nCount_RNA', 'S.Score', 'G2M.Score')
)
long <- RunPCA(long, features = VariableFeatures(object = long))
ElbowPlot(long, 50)
long <- FindNeighbors(long, dims = 1:35)
long <- FindClusters(long, resolution = 0.5)
long <- RunUMAP(long, dims = 1:35)
DimPlot(long, reduction = "umap")

cds <- as.cell_data_set(long)
cds <- cluster_cells(cds, resolution = 0.003)
p1 <- plot_cells(cds, show_trajectory_graph = FALSE)
p2 <- plot_cells(cds, color_cells_by = "cluster", show_trajectory_graph = FALSE)
wrap_plots(p1, p2)
cds <- learn_graph(cds)
plot_cells(cds, label_groups_by_cluster = FALSE, label_leaves = FALSE, label_branch_points =
FALSE)
cds <- order_cells(cds)
plot_cells(cds, color_cells_by = "pseudotime", label_cell_groups = FALSE, label_leaves =
FALSE,
    label_branch_points = FALSE)
```
